# Seasonal Differences in Fish Community Structure in the Upper Yangtze River Based on eDNA Metabarcoding: A Multi‐Dimensional Analysis

**DOI:** 10.1002/ece3.72215

**Published:** 2025-10-14

**Authors:** Yixiao Li, Lei Zhou, Renhui Luo, Zhiling Dong, Hongsen Lv, Jingning Ling, Weizhi Yao, Wenping He

**Affiliations:** ^1^ Research Center for Aquatic Biodiversity Conservation in the Upper Reaches of Yangtze River, Ministry of Agriculture and Rural Affairs, College of Fisheries Southwest University Chongqing China; ^2^ Fisheries College Ocean University of China Qingdao China; ^3^ Key Laboratory of Freshwater Fish Reproduction and Development, Ministry of Education, College of Fisheries Southwest University Chongqing China

**Keywords:** eDNA metabarcoding, fish functional groups, multidimensional diversity, seasonal variations, upper Yangtze River

## Abstract

This study used environmental DNA (eDNA) metabarcoding to monitor fish communities at 14 sites along the upper Yangtze River across four seasons, aiming to understand seasonal variations in community structure. This study reflects on the current status of fish community structure in the region by leveraging an analytical discussion on the composition of fish species and functional groups, the application of 16 multidimensional diversity indices, and the relationships between fish communities and environmental variables. A total of 120 fish species were detected. The communities predominantly consisted of species that produce sticky eggs, are sedentary, and have omnivorous diets. However, the sequence reads of species producing drifting eggs, migratory, and carnivore fish groups were higher in autumn and winter compared to spring and summer. Except for the functional evenness index (FEve), all other functional diversity indices, as well as α diversity, taxonomic diversity, and phylogenetic diversity indices, were higher in spring and summer compared to autumn and winter. This suggests that fish diversity is greater in spring and summer due to increased fish activity during the spawning season and higher eDNA shedding and detectability under warmer conditions; however, ecological niche overlap is more pronounced during these seasons. This study provides practical experience for fish monitoring based on eDNA metabarcoding technology.

## Graphical abstract

This study used eDNA metabarcoding to detect seasonal fish diversity in the upper Yangtze River, analyzing species composition and functional groups. Multiple diversity indices (α‐diversity, functional, taxonomic, and phylogenetic diversity) were employed to explore seasonal changes in fish communities. The multi‐dimensional analysis provides a comprehensive understanding of fish resources, supporting targeted conservation efforts.

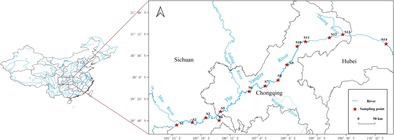

## 1 Introduction

The Upper Yangtze River, with its unique geographical features and hydrological conditions, provides habitat for numerous endemic fish species and represents one of the most important fish genetic resource reservoirs in China (Liu et al. 2019). Fishes in the upper reaches of the Yangtze River constitute a vital component of the region's fishery resources. Fish populations in the upper reaches of the Yangtze River constitute a vital component of the regional ecosystem. They contribute substantially to the basin's aquatic biodiversity and play a crucial role in maintaining the ecological structure and functionality of the river system. However, these populations are increasingly threatened by human‐induced disturbances. Key drivers include the construction of hydraulic infrastructure, the introduction and spread of alien species, and the long‐term consequences of historical overfishing. As a result, fish biomass in this region has undergone a marked decline in recent years (Jin et al. 2022; Zhou et al. 2020). Against this backdrop, monitoring fish composition and diversity, along with ecological assessments in the Upper Yangtze River basin, becomes increasingly crucial.

Traditional methods for surveying fishery resources are fraught with numerous disadvantages, such as significant habitat destruction, high costs, lengthy durations, and incomplete capture data due to the selection of net mesh sizes, which currently make it challenging to meet the needs for investigating fish composition, diversity, and resource assessment (Stoeckle et al. 2017; Yao et al. 2022; Zhu et al. 2023; Ling et al. 2022). Conversely, eDNA metabarcoding technology, characterized by its non‐invasiveness and high sensitivity, has now been widely applied in monitoring freshwater fish resources, gradually becoming the mainstream method (Fujii et al. 2019; Hervé et al. 2023; Wang, Yan, et al. 2021). The Yangtze River basin has seen numerous studies utilizing eDNA metabarcoding technology to investigate fish composition and diversity (He et al. 2022; Qu et al. 2020; Zhang, Zhang, et al. 2023). However, most of these studies have reflected the fish community structure through analyses of fish species composition and α, β diversity (Cheng et al. 2023; Jia et al. 2021; Qian et al. 2023; Shen et al. 2023). Despite the broad application of this approach, such data do not facilitate a comprehensive understanding of fish resource information.

Regarding fish composition, analyzing solely from a taxonomic perspective overlooks the unique functional roles and interconnections among fish species within ecosystems (Yang et al. 2016). Fish functional groups, composed of species within an ecosystem that share similar functions or occupy similar ecological niches (Wo et al. 2018), play specific roles in ecosystems, making the classification of fish into functional groups for analysis exceedingly critical (Halpern and Floeter 2008; Nash et al. 2016). Essentially, fish functional groups reveal the mechanisms of interaction among fish species and the adaptability of communities, aiding in our deep understanding of the roles and structural characteristics of fish within communities (Martin et al. 2016; Wang, He, and Long 2023; Gu et al. 2022).

α and β diversity are the most direct manifestations of biodiversity. Yet, they only account for species richness and abundance, overlooking other components of biodiversity (Xia et al. 2022). Functional diversity, through the lens of species‐specific functional traits, elucidates community differences, adequately reflecting the extent of similarity or divergence in species' roles within ecosystems (Zhang, Zhai, et al. 2023). Taxonomic diversity quantifies the phylogenetic relationships among species and the imbalance in taxonomic relations, showcasing the diversity of biological lineages within communities (Wang, Hu, et al. 2021). From the perspective of the evolutionary history of regional communities, phylogenetic diversity characterizes the ecological differences among species, significantly contributing to biodiversity conservation (Zhuo et al. 2024). The diversity of functional groups elucidates the relationship between fish communities and the scale of their accessible niches and habitats, indicating the richness of community functions (Ma 2021). Utilizing a multifaceted approach to quantify biodiversity allows for a more comprehensive and profound understanding of the current patterns of biodiversity, facilitating enhancements in biodiversity conservation (Weithoff et al. 2015).

This study employed eDNA metabarcoding technology to conduct fish detection across four seasons in the upper reaches of the Yangtze River. By dissecting individual fish species and fish functional group compositions and integrating various diversity models such as species α diversity, β diversity, functional diversity, taxonomic diversity, phylogenetic diversity, and functional group α diversity, a comprehensive and systematic analysis was performed on the seasonal variations of fish communities in the upper Yangtze River. This study aims to provide complete foundational data support for improving and implementing fishery resource management and species conservation policies in the Upper Yangtze River, as well as practical experience for fully utilizing eDNA metabarcoding technology in fish monitoring data.

## 2 Materials and Methods

### 2.1 Water Sample Collection and eDNA Filtration Procedures

**FIGURE 1:**
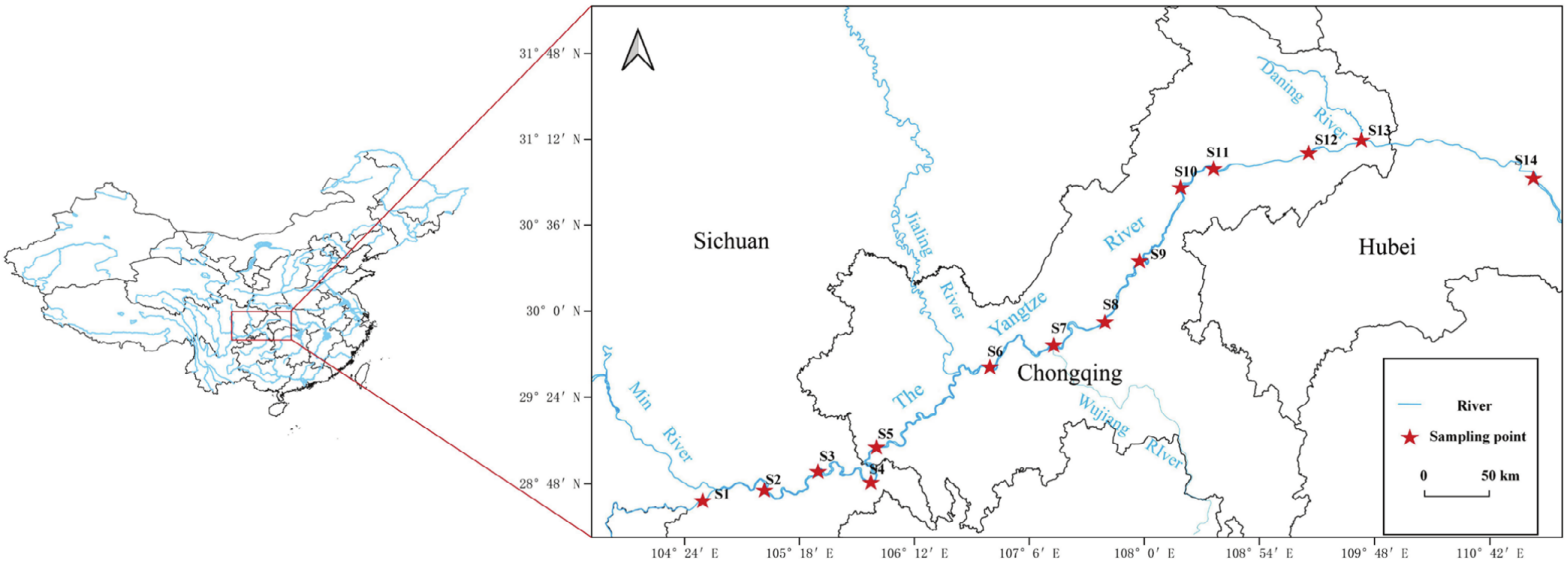
Distribution of sampling points in the upper reaches of the Yangtze River.

Water samples were collected from 14 sampling points along the main channel of the upper reaches of the Yangtze River during the autumn of October 2021 and subsequently in the winter of January 2022, spring of April 2022, and summer of July 2022 (Figure 1).

At each sampling site, surface water (0–1 m depth) was collected from the left bank, center, and right bank of the river using a 5 L water sampler at each position. The three subsamples (5 L each) were first combined and thoroughly mixed to obtain a total of 15 L of composite water. From this mixture, 10 L was randomly taken and transferred into a clean polyethylene container for subsequent analysis. All sampling tools were rinsed three times with the river water to be collected at each site prior to sampling. During sampling, new disposable masks and rubber gloves were worn at each sampling site to avoid cross‐contamination of water samples from different locations. All samples were vacuum‐filtered within 24 h using a 0.45 μm pore size polytetrafluoroethylene (PTFE) filter membrane. Three replicates were filtered at each sampling site, with each replicate consisting of 2 L of water subsampled from a single 10 L composite sample. A single negative control using sterile distilled water was included for the entire experiment to assess the presence of exogenous DNA contamination. After filtration, the filter membranes were immediately rolled with sterile tweezers, facing inward, into a cylindrical shape and placed into 10 mL centrifuge tubes, then stored at −80°C until further processing (Shu et al. 2021). Before filtration, the necessary filtering materials were disinfected with 75% ethanol and rinsed with distilled water. After each filtration step, the water buckets and filtering tools were thoroughly cleaned using a 10% sodium hypochlorite (bleach) solution to degrade any residual DNA and prevent cross‐contamination between samples.

### 2.2 Environmental Factors

Water temperature (WT), dissolved oxygen (DO), conductivity (Con), and pH were measured at each sampling site using a HACH portable multiparameter water quality analyzer. Water flow velocity (FV) and depth (Dep) were also measured with a biomimetic net mouth flow meter and a portable depth gauge, respectively.

### 2.3 EDNA Extraction, High‐Throughput Sequencing, and Data Processing

DNA enriched on the filter membranes was extracted using the HiPure Water DNA Kit produced by Magen. The extracted DNA samples were verified by 1% agarose gel electrophoresis. PCR amplification was performed using the extracted DNA samples as templates and Mifish‐U primers (Miya et al. 2015). The first‐round PCR was performed using TransGen AP221‐02: TransStart Fastpfu DNA Polymerase in a 20 μL reaction volume, consisting of 4 μL of 5× FastPfu Buffer, 2 μL of 2.5 mM dNTPs, 0.8 μL of forward primer (5 μM), 0.8 μL of reverse primer (5 μM), 4 μL of FastPfu Polymerase, 10 ng of template DNA, and ddH_2_O to a final volume of 20 μL. Each sample was amplified in triplicate to ensure reproducibility. After PCR amplification, the products from the same sample were pooled, and the concentration was assessed using 2% agarose gel electrophoresis. Bands of the desired size were excised from the gel and purified using the AxyPrep DNA Gel Recovery Kit (AXYGEN), followed by Tris HCl elution. The purified PCR products were then subjected to a second round of 2% agarose gel electrophoresis to verify their purity. Samples producing bands of the desired brightness were sent for further processing. Sequencing was performed using the Illumina PE250 platform. Effective sequences for all eDNA samples were obtained via barcode splitting, followed by rigorous quality control, filtering, and Operational Taxonomic Unit (OTU) clustering. Raw reads were processed as follows: reads with a mean quality score below 20 over a 10 bp sliding window were trimmed, and sequences shorter than 50 bp after trimming were discarded. Paired‐end reads were merged based on an overlap of at least 10 bp with a mismatch ratio ≤ 0.2. Based on barcodes and primers, merged reads were demultiplexed, with barcode mismatches not allowed and primer mismatches allowed up to 2 bases. Chimeric sequences were removed using Usearch by combining de novo and reference‐based approaches with the gold database. OTU clustering was performed using the UCLUST algorithm (v10) at a 97% sequence similarity threshold. Representative sequences of each OTU were taxonomically annotated using BLASTn (*e*‐value ≤ 1e‐10) against a freshwater fish mitochondrial reference database provided by Shanghai Lingen Biotechnology Co. Ltd. When multiple candidate species were matched, the species with the highest similarity was annotated. The sequencing work was completed at Shanghai Lingen Biotechnology Co. Ltd., ultimately producing an OTU sequence reads table.

Based on the OTU sequence analysis results from each sampling site, OTUs annotated as “Unclassified” were removed, as they could not be assigned to any known taxonomic group. Additionally, OTUs detected in the negative control were excluded when their read counts in the samples were less than or equal to those observed in the negative control, to minimize potential contamination while retaining biologically relevant signals. Information on fish species not distributed in the upper reaches of the Yangtze River and marine fish was removed based on the China Animal Scientific Database (http://www.zoology.csdb.cn/) and “The Fishes of Sichuan” (Ding 1994).

### 2.4 Data Analysis and Visualization

#### 2.4.1 Fish Composition

In R4.3.1, the “venn” (v0.1.10; Yan 2023) and “ggplot2” (Wickham 2016) packages were used to visually display the number of fish species and sequence reads across different seasons.

Regarding functional groups, considering aspects such as reproduction, nutrition, phenotype, and ecological habits of fish, 7 categorical and 2 continuous functional traits were selected for functional group clustering (Table S1). Functional trait data for fish were collected through a literature review, the China Animal Scientific Database (http://www.zoology.csdb.cn/), and “The Fishes of Sichuan.” If the trait information for the target species was unavailable in the collected literature, traits of species within the same or similar genus were referenced or noted as “unknown.” Fish species classified as “unknown” were included in the functional group clustering analysis as a separate category. These species were assigned to appropriate functional groups based on their ecological and phenotypic characteristics as available, with their traits treated as “unknown” during the analysis.

The “gowdis” function of the FD package in R4.3.1 was used to calculate the Gower distance matrix between species. Clustering was then performed using the ward.D method of the “hclust” function, and the “ggtree” (v3.4.1; Yu et al. 2017) package was used to visualize the clustering tree. Species were grouped into functional groups based on a certain distance, forming combinations of similar functional species groups.

#### 2.4.2 Diversity Indices

Relevant diversity indices were selected to describe various aspects of diversity, with seasonal differences visualized using box plots. Sequence read data were normalized by rarefaction, and rarefied sequence reads after normalization were used as the basis for calculating diversity indices. The Kruskal–Wallis test was applied to assess the statistical significance of seasonal differences in diversity indices. Visualizations were performed in R 4.3.1.

##### 2.4.2.1 Species and Functional Group α‐Diversity

The Margalef index, Shannon index, Simpson index, and Pielou's evenness index (Wang, He, and Wang, Wang, et al. 2023) were selected to describe the richness and evenness of fish species and functional groups each season. The alpha diversity markers of species were SP Margalef, SP Shannon, SP Simpson, and SP Pielou, and the alpha diversity markers of functional groups were FG Margalef, FG Shannon, FG Simpson, and FG Pielou. Calculations were performed using the rarefied sequence reads as input. Calculations were performed using the “picante” (v1.8.2; Kembel et al. 2010) and “vegan” (v2.6‐4; Oksanen et al. 2022) packages in R4.3.1.

$$D_{\text{margalef}}=\frac{S-1}{\ln N}$$

$$H_{\text{shannon}}=-\sum W_{i}\ln W_{i}$$

$$D_{\text{simpson}}=1-\sum W_{i}^{2}$$

$$E_{\text{pielou}}=\frac{H_{\text{shannon}}}{\ln S}$$

where *S* is the number of fish species, *N* is the total number of sequences, and *W*_*i*_ is the proportion of the rarefied sequence reads of the *i*th fish species to the total sequence abundance.

##### 2.4.2.2 Functional Diversity

Referring to the species functional traits collected in “2.6.1,” functional richness (FRic), functional evenness (FEve), functional divergence (FDiv), functional dispersion, and Rao's quadratic entropy index (FD_Q_) were selected to quantify functional diversity. Species‐based functional traits and rarefied sequence reads were used. Calculations were performed using the “FD” (v1.0‐12.3; Laliberté and Legendre 2010; Laliberté et al. 2014) package in R4.3.1 (Gong et al. 2024). In this study, species traits were derived from species identified through eDNA sequencing, and the rarefied sequence reads obtained from eDNA data were used in place of traditional species abundance counts.

Functional richness index indicates the extent to which a community's species occupy the functional space, calculated as follows (Villéger et al. 2008):

$${\mathrm{FR}}_{ic}=\frac{{\mathrm{SF}}_{ic}}{R_{c}}$$

where SF_*i*c_ indicates the ecological niches occupied by all species in the community, which are determined based on species identified through environmental DNA (eDNA) data, *R*_c_ indicates the absolute values of the traits.

Functional evenness measures the uniform distribution of functional traits within the functional space, calculated as follows (Villéger et al. 2008):

$${\mathrm{EW}}_{l}=\frac{\text{dist}\left(i,j\right)}{W_{i}+W_{j}}$$

$${\mathrm{PEW}}_{l}=\frac{{\mathrm{EW}}_{l}}{\sum_{l=1}^{S-1}{\mathrm{EW}}_{l}}$$

$${\mathrm{FE}}_{\mathrm{ve}}=\frac{\sum_{l=1}^{S-1}\min\left({\mathrm{PEW}}_{l},\frac{1}{S-1}\right)-\left(\frac{1}{S-1}\right)}{1-\frac{1}{S-1}}$$

where EW_*l*_ is the evenness weight, dist (*i*, *j*) is the Euclidean distance between species *i* and *j*, *W*_*j*_ is the relative abundance of species *j*, *l* is the branch length, and PEW_*l*_ is the branch length weight.

Functional divergence is an indicator measuring the variation of functional traits, reflecting resource differentiation and the degree of competition between species, calculated as follows (Villéger et al. 2008):

$$g_{k}=\frac{1}{S}\times \sum_{i=1}^{S}x_{\mathrm{ik}}$$

$${\mathrm{dG}}_{i}=\sqrt{\sum_{k=1}^{T}{\left(x_{\mathrm{ik}}-g_{k}\right)}^{2}}$$

$$\bar{\mathrm{dG}}=\frac{1}{S}\times \sum_{i=1}^{S}{\mathrm{dG}}_{i}$$

$$∆d=\sum_{i=1}^{S}W_{i}\times \left({\mathrm{dG}}_{i}-\bar{\mathrm{dG}}\right)$$

$$∆\left|d\right|=\sum_{i=1}^{S}W_{i}\times \left|\left({\mathrm{dG}}_{i}-\bar{\mathrm{dG}}\right)\right|$$

$${\mathrm{FD}}_{\mathrm{iv}}=\frac{∆d+\bar{\mathrm{dG}}}{∆\left|d\right|+\bar{\mathrm{dG}}}$$

where *x*_*ik*_ is the trait value *k* of species *i*, *g*_*k*_ is the centroid of trait *k*, *T* is the number of traits, Δ|*d*| is the average distance of species *i* from the centroid, and Δ*d* is the dispersion weighted by relative abundance.

Functional dispersion represents the average distance of individual species in a multidimensional trait space to the centroid of all species; it interprets species richness by moving the centroid's position towards more abundant species and weights individual distances by relative abundance, calculated as follows (Laliberté and Legendre 2010):

$$c=\left[c_{i}\right]=\frac{\sum a_{j}x_{\mathrm{ij}}}{\sum a_{j}}$$

$${\mathrm{FD}}_{\text{is}}=\frac{\sum a_{j}z_{j}}{\sum a_{j}}$$

where *a*_*j*_ is the abundance of species *j*, *z*_*j*_ is the distance of species *j* in two‐dimensional space to the weighted centroid *c*, and *x*_*j*_ represents the position of species *j* in two‐dimensional space.

Rao's quadratic entropy index reflects the complementarity of ecological niches among species, quantitatively representing the heterogeneity of trait values in the community. Higher functional heterogeneity suggests weaker effects of niche overlap and less resource competition, calculated as follows (Botta‐Dukát 2005):

$$d_{\mathrm{ij}}=\frac{1}{n}\sum_{k=1}^{n}{\left(X_{\mathrm{ik}}-X_{\mathrm{jk}}\right)}^{2}$$

$${\mathrm{FD}}_{Q}=\sum_{i=1}^{S}\sum_{j=1}^{S}d_{\mathrm{ij}}P_{i}P_{j}$$

where *S* is the total number of species in the sample; *P*_*i*_, *P*_*j*_ are the relative abundances of species *i*, *j* in the community; *d*_*ij*_ is the interspecific distance or difference between species *i* and *j*, *n* is the number of traits, *X*_*ik*_ is the trait value *k* of species *i*, and *X*_*jk*_ is the trait value *k* of species *j*.

##### 2.4.2.3 Taxonomic Diversity

Indices selected for taxonomic diversity analysis mainly include the average taxonomic distinctness index (Δ+) and the variation in taxonomic distinctness index (Λ+). Δ+ reflects the phylogenetic distance among species within a community; Λ+ reflects the uniformity of taxonomic relationships among community species (Clarke and Warwick 2001, 1998). Calculations were performed using PRIMER 7 (trial version), with fish classification status determined at four taxonomic levels: order, family, genus, and species. Different levels of taxonomic diversity were given equal weight (Li et al. 2020; Xiong et al. 2024).

$$\Delta^{+}=\frac{\sum \sum_{i\neq j}\omega_{\mathrm{ij}}}{s\left(s-1\right)}$$

$$\Lambda^{+}=\frac{\sum \sum_{i\neq j}{\left(\omega_{\mathrm{ij}}-\Delta^{+}\right)}^{2}}{s\left(s-1\right)}$$

where *ω*_*ij*_ is the path length between the *i*th and *j*th species in the taxonomic tree, *S* is the number of species.

##### 2.4.2.4 Phylogenetic Diversity

The phylogenetic diversity (PD) index was selected to describe the level of phylogenetic diversity. This index represents the total length of all the shortest path branches connecting all species on the community's phylogenetic tree (Yang 2023). Calculations were performed using PRIMER 7 (trial version).

#### 2.4.3 Beta Diversity

To investigate the seasonal variations in the structure and function of fish communities, this study analyzes species composition and functional group composition, using Non‐metric Multidimensional Scaling (NMDS). In the analysis of functional groups, the sequence reads of all species within each functional group were summed to form a new matrix. This matrix represents the sequence reads of each functional group across the four seasons and was used as input for the NMDS analysis of functional groups. To assess whether seasonal differences in species composition and functional group composition were statistically significant, a PERMANOVA (Permutational Multivariate Analysis of Variance) was performed based on Bray–Curtis dissimilarity. The Bray–Curtis distance is computed using the “vegan” (v2.6‐4; Oksanen et al. 2022) package in R version 4.3.1, and clustering is performed using the Unweighted Pair Group Method with Arithmetic Mean (UPGMA). Visualization of the results is conducted using the “ggplot2” (Wickham 2016) package.

#### 2.4.4 Correlation Analysis

Pearson correlation analysis between diversity indices and environmental factors was performed using the “rcorr” function from the “Hmisc” (v5.2‐1; Harrell 2024) package in R 4.3.1. The significance of correlations was assessed via two‐tailed *t*‐tests provided by the function. The results were visualized with the “corrplot” (v0.95; Wei and Simko 2024) package, where significant correlations (*p* < 0.05) were marked with asterisks. Initially, detrended correspondence analysis (DCA) was performed using the “vegan” package to determine the axis lengths, followed by the selection of redundancy analysis (RDA) or canonical correspondence analysis (CCA) to explore the impacts of environmental factors on the composition of fish species and functional groups.

## 3 Results

### 3.1 Species Composition

**FIGURE 2:**
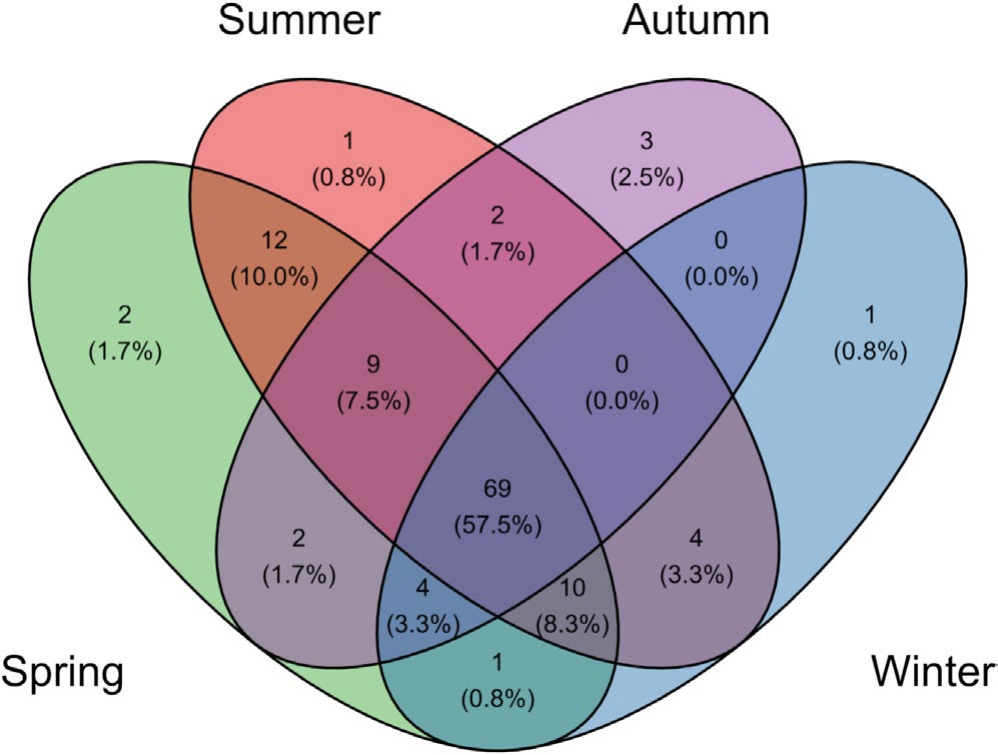
Wayne diagram of species in the upper reaches of the Yangtze River in the fourth quarter.

**FIGURE 3:**
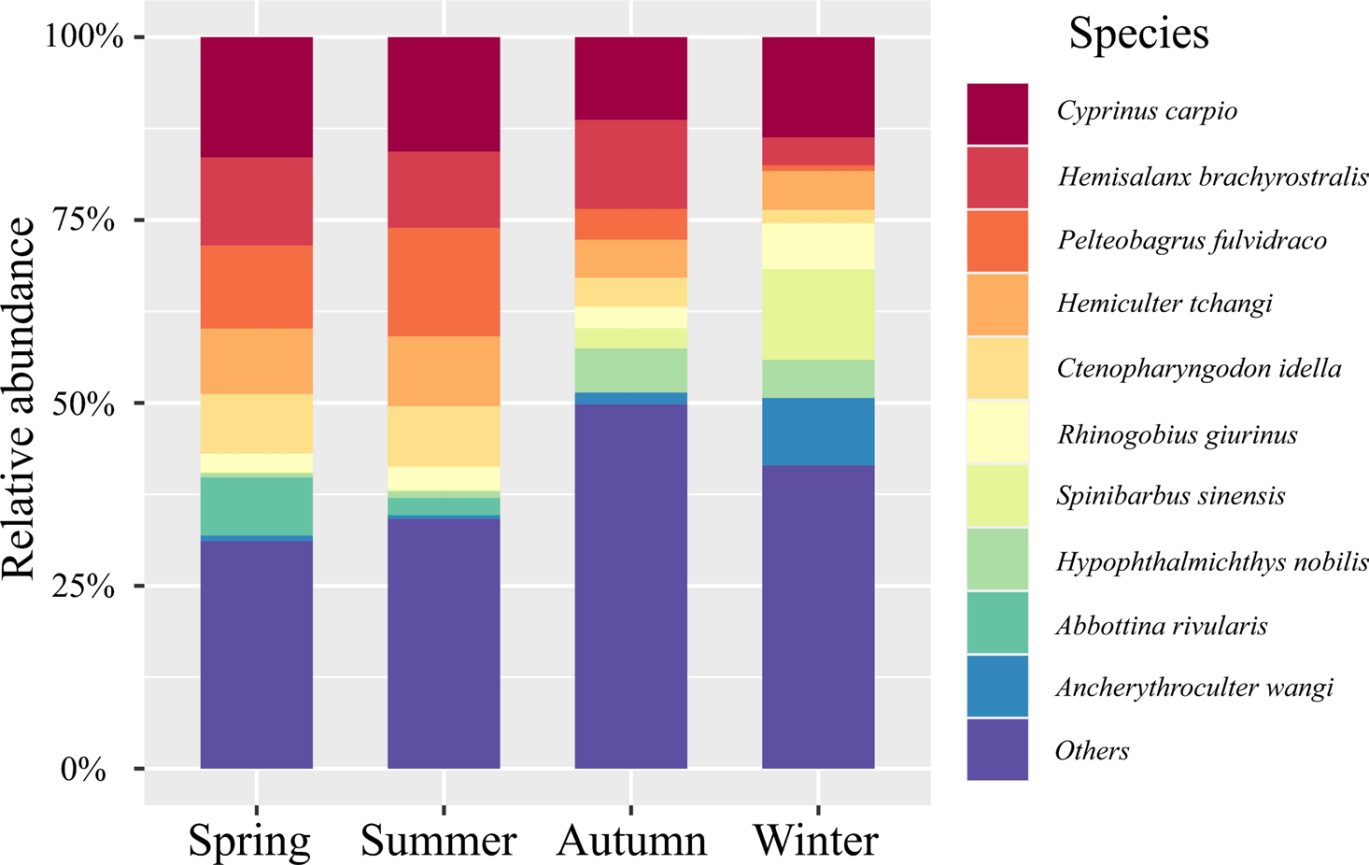
Stacked column chart of percentage of top 10 species in relative sequence abundance in the upper reaches of the Yangtze River in the fourth quarter.

In this study, a total of 3,963,137 sequence reads were obtained from all seasonal samples, including 1,178,184 in spring, 952,039 in summer, 1,130,627 in autumn, and 702,287 in winter. A total of 120 fish species were detected throughout the four seasons of the study (Table S2), classified into 7 orders, 20 families, and 79 genera, including the unidentified species level of *Oreochromis* spp. Among these, Cyprinidae was the most represented, with 69 species accounting for 57.5% of the total species count, followed by the Cobitidae family with 10 species, comprising 8.3% of the total species count. Among the species detected, 10 species are designated as national key protected species, specifically *Acipenser dabryanus* , *Leptobotia elongata* , *Leptobotia rubrilabris* , *Myxocyprinus asiaticus* , *Coreius guichenoti* , *Gobiocypris rarus* , *Procypris rabaudi* , *Rhinogobio ventralis* , *Schizothorax davidi* , and *Liobagrus kingi* . Additionally, 21 species endemic to the upper reaches of the Yangtze River and 11 alien species were identified, representing 17.5% and 9.2% of the total species count, respectively. The number of fish species detected and their sequence reads varied across the four quarters (Figures 2 and 3). There were 69 fish species that appeared consistently in spring, summer, autumn, and winter, with *Cyprinus carpio* , *Hemisalanx brachyrostralis* , and *Hemiculter tchangi* showing high sequence reads throughout all seasons. The species count for spring was identified as 109 species across 7 orders, 19 families, and 77 genera; for summer, 107 species across 7 orders, 19 families, and 74 genera; for autumn, 89 species across 7 orders, 17 families, and 65 genera; and for winter, 89 species across 7 orders, 19 families, and 70 genera.

**FIGURE 4:**
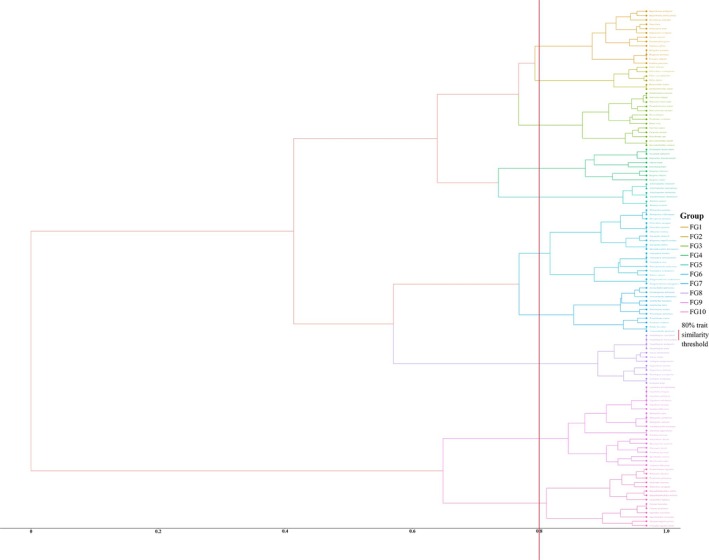
Fish functional group clustering tree.

**FIGURE 5:**
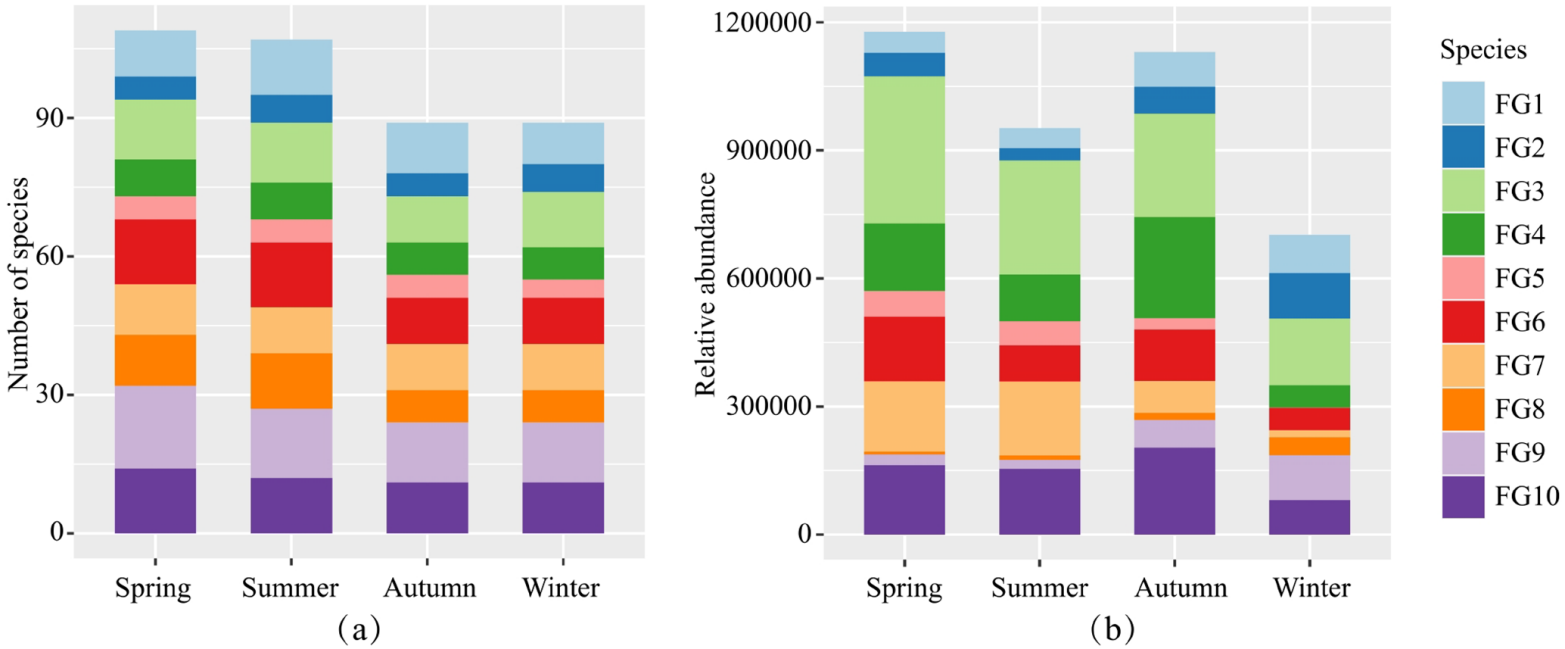
Percentage stacking column chart of 10 functional groups in the fourth quarter. (a) Based on species number. (b) Based on sequence abundance.

In this study, the fish species detected in the upper reaches of the Yangtze River were classified into 10 functional groups based on 80% similarity in functional traits (Figure 4; Table S3). The main functional traits of these groups are outlined in Table 1. Sticky eggs, sedentary, and omnivorous feeding are common traits among many fish within most functional groups, indicating that these traits are characteristic of most fish in the upper Yangtze River. Drifting eggs, migratory, and carnivorous feeding also serve as common traits among many fish within a minority of functional groups. The proportion of herbivorous and filter‐feeding fish is deficient; therefore, these traits were not identified as common traits of fishes within any functional group. Seasonal variations in the composition of functional groups, in terms of species count, were not significant, but some differences were observed when considering sequence abundance. FG3 constituted the most significant proportion in all four seasons. Compared across seasons, the composition of functional groups in spring and summer showed similarity, with FG3 and FG7 having higher proportions. In autumn, FG4 had a higher proportion, while in winter, FG1, FG2, FG8, and FG9 were more prevalent (Figure 5).

**TABLE 1:** Functional traits of various functional groups (80% of the species in the group are the traits).

Functional group	Functional traits
FG1	Sticky eggs, Sedentary, Omnivory, Quiet slow flow, Compressed
FG2	Sticky eggs, Sedentary, Carnivore, Pelagic, Compressed, Subsuperior or Superior
FG3	Sedentary, Omnivory, Laterally Flat‐Shaped, Terminal
FG4	Carnivore, Terminal
FG5	Other egg types, Sedentary, Omnivory, Quiet slow flow, Oviform
FG6	Sedentary, Omnivory, Demersal, Cylindrical, Inferior
FG7	Sedentary, Omnivory, Mesopelagic, Inferior
FG8	Sticky eggs, Sedentary, Carnivore, Other body shapes
FG9	Drifting eggs, Migratory, Omnivory, Rapid flow, Inferior
FG10	Drifting eggs, Migratory

### 3.2 Diversity Indices

**FIGURE 6:**
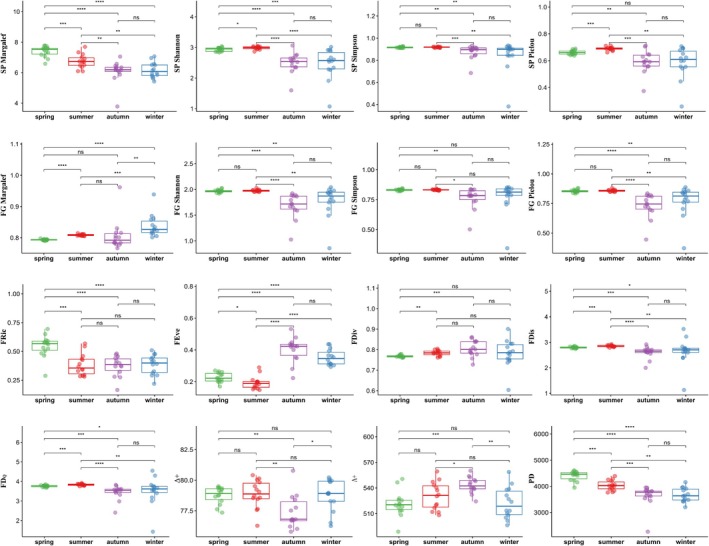
Box chart of seasonal differences in fish diversity indices in the upper reaches of the Yangtze River. The significance levels are defined as follows: ****p ≤ 0.001, ***0.001 < *p* ≤ 0.01, **0.01 < *p* ≤ 0.05, *0.05 < *p* ≤ 0.1, not significant (ns) = *p* > 0.1.

Species α‐diversity showed clear seasonal variation, with all four indices significantly higher in spring and summer than in autumn and winter (Figure 6). For functional group α‐diversity, the Margalef index was highest in winter. In contrast, the Shannon and Pielou indices were higher in spring and summer than in autumn and winter, with the Simpson index showing significant differences only between spring and summer against autumn. Functional diversity indices showed that FRic was significantly higher in spring than in other seasons, FEve was significantly higher in autumn and winter than in spring and summer, and FDis and FDQ were significantly higher in spring and summer than in autumn and winter, with FDiv only showing differences between spring and the other seasons. Taxonomic diversity indices showed significant differences between autumn and the other three seasons, with Δ+ being the lowest in autumn and Λ+ being the highest. Phylogenetic diversity (PD) followed the pattern of spring > summer > autumn > winter, though the difference between autumn and winter was not significant.

### 3.3 NMDS Analysis

**FIGURE 7:**
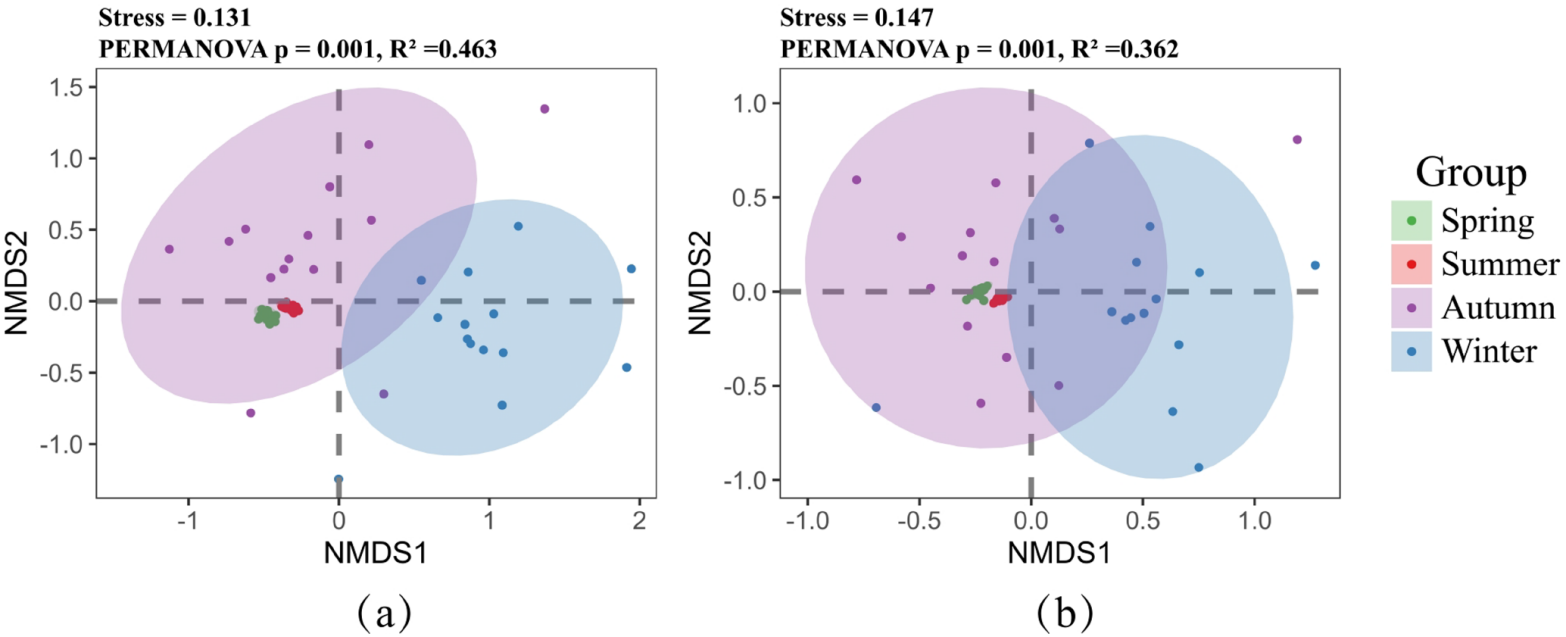
NMDS analysis results of species composition (a) and functional group composition (b) of fishes in four seasons in the upper reaches of the Yangtze River.

NMDS analysis revealed clear seasonal differences in fish community composition (Figure 7). The stress values of 0.131 for species composition and 0.147 for functional group composition indicate an acceptable level of ordination accuracy. While NMDS provides a visual representation of dissimilarities, statistical testing using PERMANOVA revealed significant seasonal differences (*p* = 0.001), with *R* ^2^ values of 0.463 for species composition and 0.362 for functional group composition. These results confirm that fish communities in the upper Yangtze River exhibit distinct seasonal patterns in both taxonomic composition and functional traits. Distinct seasonal clustering of samples indicates clear temporal separation and ecological heterogeneity (Figure 7a,b).

### 3.4 Pearson Correlation Analysis

**FIGURE 8:**
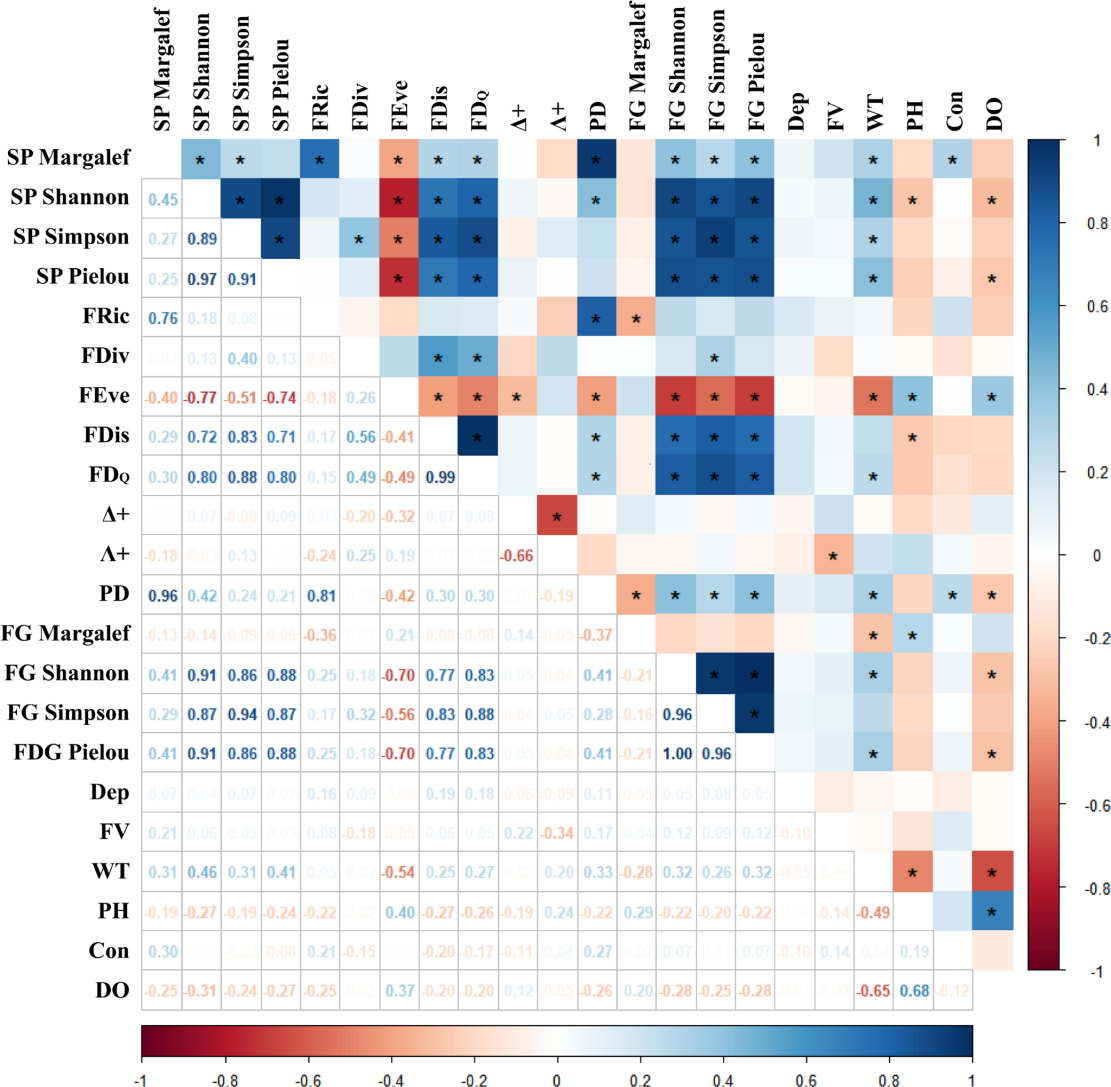
Presents the Pearson correlation matrix for the diversity indices of fish species in the upper Yangtze River and environmental factors (where “Dep” represents depth, “FV” represents flow velocity, “WT” is water temperature, “Con” is conductivity, and “DO” is dissolved oxygen).

Statistically significant correlations (*p* < 0.05) were observed among several diversity indices (Figure 8). SP Shannon, SP Simpson, SP Pielou, FG Shannon, FG Simpson, FG Pielou, FEve, FDis, and FD_Q_ showed significant positive correlation (*p* < 0.05) with one another, while FEve exhibited a significant negative correlation (*p* < 0.05) with most of these indices. A significant positive correlation (*p* < 0.05) was also found among SP Margalef, FRic, and PD, supporting the close link between species richness and functional or phylogenetic diversity. In contrast, a significant negative correlation (*p* < 0.05) was observed between the indices Δ+ and Λ+. Regarding environmental factors, WT, pH, and DO showed significant correlations (*p* < 0.05) with multiple diversity indices, among which WT was associated with the greatest number of indices. Among all diversity indices, FEve was the most strongly influenced by environmental factors, indicating that its variation is more sensitive to environmental changes.

### 3.5 RDA Analysis Results

**FIGURE 9:**
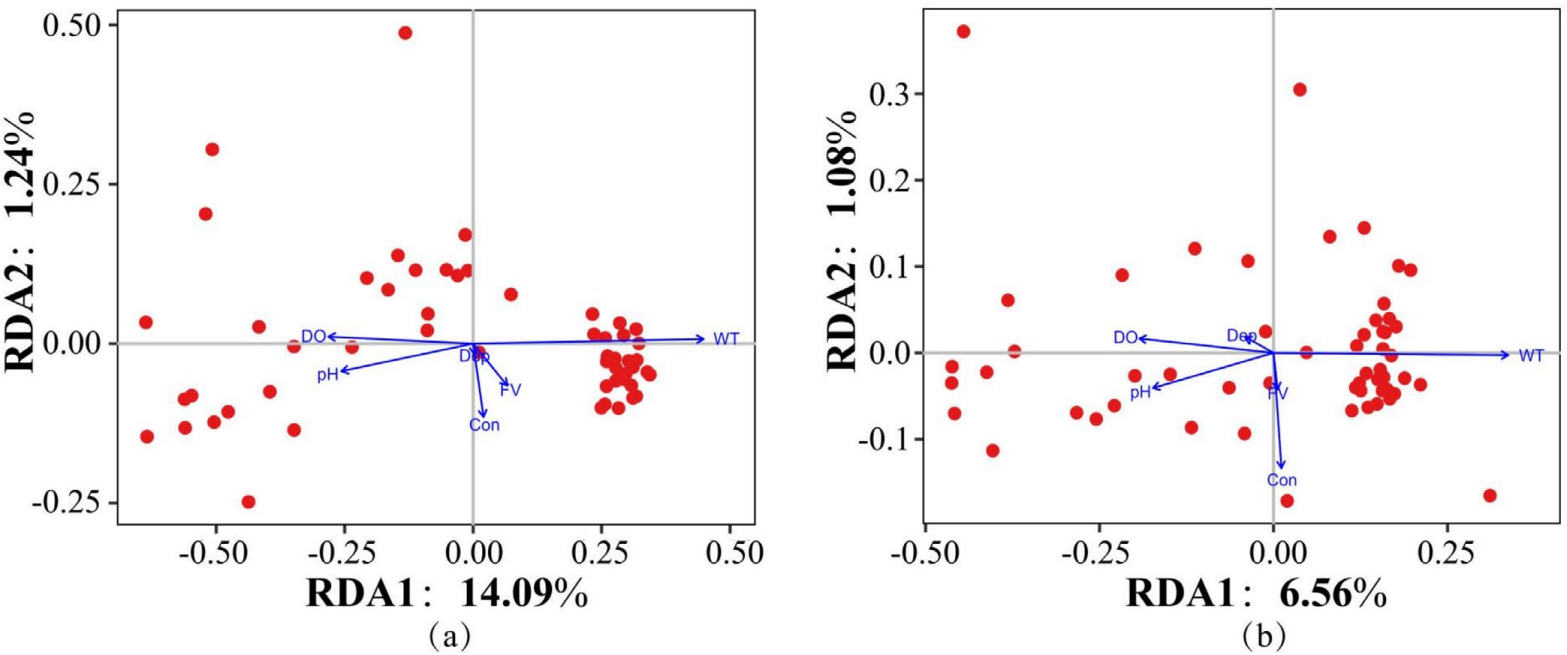
Presents the RDA analysis of fish species composition (a) and functional group composition (b) with environmental factors in the upper Yangtze River.

The results of the DA analysis indicated that the length of the first axis for species composition was 3.5429, and for functional group composition, it was 1.6251. Consequently, the Redundancy Analysis (RDA) method was selected for subsequent analyses (Figure 9). WT (*R* ^2^ = 0.6855, *p* < 0.001), Con (*R* ^2^ = 0.3634, *p* < 0.001), DO (*R* ^2^ = 0.2666, *p* < 0.001), and pH (*R* ^2^ = 0.2904, *p* = 0.003) had significant impacts on fish species composition, while WT (*R* ^2^ = 0.6290, *p* < 0.001), Con (*R* ^2^ = 0.5397, *p* < 0.001), DO (*R* ^2^ = 0.2088, *p* < 0.001), and pH (*R* ^2^ = 0.2302, *p* = 0.005) significantly influenced the composition of fish functional groups (Figure 9a,b).

## 4 Discussion

### 4.1 Fish Species Composition and Functional Group Composition

The eDNA method not only provides information on species richness and distribution but also enables the monitoring of environmental changes and their impacts on fish communities, thereby supporting ecological conservation and fisheries management. Consequently, an increasing number of studies are employing eDNA metabarcoding technology to assess the status of fisheries resources, providing a scientific foundation for sustainable management (Banerjee et al. 2021; Xiong et al. 2022). Utilizing eDNA metabarcoding technology, we detected 120 fish species in the upper reaches of the Yangtze River across four seasons; Cyprinidae accounted for a large proportion. This finding aligns with the results of numerous previous studies (Gao et al. 2019, 2013; He et al. 2018). The composition of fish species exhibited seasonal variations (Li, Jiang, et al. 2023; Suzuki et al. 2022). In this study, species diversity was higher in spring and summer compared to autumn and winter. Previous studies have indicated that eDNA concentrations are lower in autumn and winter than in spring and summer (Buxton et al. 2018; Troth et al. 2021). This is likely due to increased fish activity during the breeding season, which promotes the release of eDNA (Hayami et al. 2020). This difference is primarily driven by changes in the population of Cypriniformes, with approximately 20 more species of fish observed in spring and summer than in autumn and winter. Notably, changes in Cypriniformes account for over 50% of this variation. Seven out of the top 10 species by abundance belong to the order Cypriniformes, and their sequence reads show significant seasonal differences (Figure 3).

The NMDS analysis revealed that fish communities in spring and summer exhibited tighter clustering, whereas those in autumn and winter displayed greater dispersion (Figure 7). This indicates a higher degree of community homogeneity during the warmer seasons and increased heterogeneity in colder periods. Such seasonal differences may be attributed to synchronized spawning activities and more stable environmental conditions in spring and summer, which promote similar species composition across sampling sites. In addition, this seasonal pattern may be influenced by hydrological dynamics. During the wet season (spring and summer), elevated water levels can lead to the formation of floodplains, which enhance lateral connectivity between the main river channel and adjacent habitats. This expanded connectivity facilitates fish movement across different habitats, leading to a more homogeneous distribution of species (Thomaz et al. 2007; Zhang, Wu, et al. 2019). By contrast, during the dry season (autumn and winter), falling water levels reduce hydrological connectivity and eliminate floodplain habitats, resulting in increased habitat fragmentation. These physical barriers may restrict fish dispersal and contribute to the observed spatial heterogeneity in community composition (Branco et al. 2017; Bednarek and Mołoniewicz 2023).

There is a distinct seasonal variation in the composition of functional groups (Figure 5), with fish in the upper Yangtze River predominantly characterized by sticky eggs production, sedentariness, and omnivorous feeding habits, consistent with the bulk of research (Cheng et al. 2023; Yi and Wang 2009; Li et al. 2013). The obstruction caused by cascading hydropower dams in the Yangtze River basin prevents migratory fish species that produce drifting eggs from reaching their spawning grounds. In contrast, the reproduction of most benthic fish species that lay adhesive eggs is minimally affected (Lin et al. 2016). Furthermore, the periodic impoundment of dams may provide a broader range of food resources for fish (Xie et al. 2019), thereby enabling fish species with these characteristics to occupy a larger ecological niche within the ecosystem. FG6 and FG7 are primarily composed of sedentary, omnivorous, subterminal‐mouthed fish species, which feed on benthic organisms and exhibit seasonal variations. FG7 shows significantly higher sequence reads in spring and summer than in autumn and winter, whereas FG6's seasonal variation is less pronounced. This difference is attributed to other characteristics of the functional groups. Post‐operation of the cascading hydropower stations leads to slower water flow and sediment deposition, disrupting suitable living conditions for benthic organisms serving as fish food, thus reducing biomass. FG6, primarily comprising cylindrical, bottom‐dwelling Cobitidae species, is well‐adapted to sediment substrates and has a broad diet; in contrast, FG7 consists of mesopelagic fish, most of which prefer living in gravel substrates with a narrower diet focusing on algae scraping (Su et al. 2015). During the flood season in April and July, increased flow rates remove bottom sediments, creating suitable habitats for FG7. In contrast, during autumn in October and winter in January, most fish offspring develop into juvenile stages, and the ecological niches of carnivorous groups FG2, FG4, and FG8 broaden due to a broader diet, increasing their sequence reads within functional groups. FG4 is comprised of fish species from the orders Perciformes and Salmoniformes; within the Perciformes, species such as *Channa argus* , *Siniperca chuatsi* , and others are included, while the Salmoniformes primarily consist of several species within Salangidae, which tend to hibernate in deep water with reduced feeding activity during winter, while the latter's autumn spawning population dies after spawning (Yang et al. 2012; Gong et al. 2010), leading to a decrease in FG4 after a peak in autumn, while FG2 and FG8 fill the ecological niche left by FG4, resulting in continuous population growth. The migratory fish groups FG9 and FG10, which produce drifting eggs, are more abundant in autumn and winter than in spring and summer breeding seasons, possibly due to our sampling locations not being in their spawning areas.

### 4.2 Fish Diversity Analysis

Significant seasonal variations in fish diversity were observed in the upper reaches of the Yangtze River, with diversity generally higher in spring and summer than in autumn and winter. Species alpha diversity indicated that species evenness and richness levels during the spring and summer seasons were superior to those in autumn and winter, aligning with findings from Dai et al. (2022). Typically, the Yangtze River basin experiences higher precipitation levels in April and July, marking the flood season with increased flow rates and abundant food resources, along with higher water temperatures conducive to fish reproduction and migration. Additionally, an increase in population supplementation during these seasons leads to richer fish resources; conversely, October and January represent the dry season, characterized by reduced flow rates, lower water temperatures, and comparatively diminished food resources and fish populations (Tian et al. 2021). At the same time, gamete release into the aquatic environment during fish reproduction, along with intensified metabolism triggered by active feeding behaviors, increases the source of fish DNA molecules in the water, thereby enhancing detection rates (Dong 2023). Thus, functional group alpha diversity also tends to be higher in spring and summer than in autumn and winter. An anomaly in FG Margalef diversity arises due to the uniform presence of 10 functional groups (FG1–FG10) across all seasons, resulting in a paradoxically higher Margalef index during winter, the season with the lowest total sequence reads. Each diversity index emphasizes different aspects and has its limitations, leading most studies to evaluate fish communities using multiple alpha diversity indices (Jia et al. 2021; Wang et al. 2022; Dong et al. 2023).

In this study, the FRic index was highest in spring, as closely related species typically exhibit more similar functions. With 77 genera of fish detected in spring, surpassing the other three seasons, the overall ecological niche of the fish community expanded, elevating the FRic index (Mason et al. 2005). The FEve index was highest in autumn and winter, possibly due to increased abundance in non‐dominant functional groups such as FG2, FG4, FG8, FG9, and FG10, primarily composed of aggressive carnivorous and migratory fish species, enhancing the community's locomotion and foraging capabilities, thereby improving utilization rates of habitats and prey organisms (He et al. 2023). The FDiv index was lowest in spring, partly because of the high dominance of certain species (Figure 4), with the top 10 species in spring exhibiting the highest sequence reads across all seasons, leading to some ecological niches being oversaturated and intense intraspecific competition (Shuai et al. 2017). The FDis and FD_Q_ indices were higher in spring and summer, indicating greater trait diversity within the fish community during these seasons, higher complementarity in resource utilization among species, lower interspecific competition, and less niche overlap (Valencia et al. 2015).

Among taxonomic diversity indices, the average taxonomic distinctness index Δ+ was significantly lower in autumn than in other seasons, while the variation in taxonomic distinctness index Λ+ was significantly higher, indicating fewer species and closer phylogenetic relationships among fish communities in autumn, but with lower evenness in taxonomic relationships (Wang, Hu, et al. 2021). The PD index suggests that phylogenetic diversity of the fish communities in the upper Yangtze River follows a seasonal pattern of spring > summer > autumn > winter.

Studies have shown a significant correlation between phylogenetic diversity (PD) index and species richness (Crandall et al. 2000; Qian et al. 2017), as well as a highly significant positive relationship between species richness and functional richness (Zhang, Zhai, et al. 2023). A significant positive correlation among SP, Margalef, PD, and FRic indices reaffirms these observations (Figure 8).

This study also found a significant correlation among nine diversity indices, namely FEve, FDis, FD_Q_, SP Shannon index, SP Simpson, SP Pielou, FG Shannon, FG Simpson, and FG Simpson. Specifically, FEve exhibited a negative correlation with the other eight indices, while the remaining eight indices were positively correlated with one another. The FDiv index appears relatively independent. Δ+ and Λ+ demonstrate a significant negative correlation, consistent with findings from a previous study (Shang et al. 2023), where groups with lower Δ+ exhibited higher Λ+. Different types of diversity indices provide various insights into ecosystem status. Using a comprehensive approach to evaluate ecological integrity allows for a more complete understanding of ecosystem structure and function and its response to major environmental drivers, facilitating a more accurate appreciation of true ecosystem diversity (Gallardo et al. 2011).

### 4.3 Impact of the Environment on Fish Community Structure

The seasonal distribution of fish community structure is primarily influenced by water temperature and fish growth and reproductive activities, among other factors (Littlefair et al. 2021). Water temperature is considered one of the main factors affecting fish communities in several studies (Lin et al. 2021; Guo et al. 2023), and in our research, it was the factor most strongly correlated with both fish composition and diversity, showing a positive relationship with most fish diversity indices. In studies of fish community structure based on eDNA metabarcoding technology, the impact of water temperature extends beyond direct effects on the fish, influencing the release of eDNA by active fish and the degradation of eDNA in the water (Coble et al. 2019). Research indicates that fish growth, reproduction, feeding intensity, metabolism, and stress responses are significantly influenced by water temperature, thereby affecting fish diversity (Duffy et al. 2016; Morita et al. 2010; Sandersfeld et al. 2017). It can be inferred that water temperature may be a major driver of eDNA distribution, as the feeding, movement, and reproductive capabilities of fish gradually increase with rising water temperatures, accelerating the shedding of eDNA and leading to higher eDNA concentrations in the water.

pH and dissolved oxygen (DO) also show significant correlations with fish community structure, generally displaying negative correlations with most diversity indices. A pH range of 6.5–9 is considered completely normal for fish survival and reproduction (Mustapha and Atolagbe 2018), and the pH range measured in this study was 7.39–8.63. It is speculated that the impact of pH on the results is due to higher pH values in autumn and winter, which increase the decay rate of eDNA (Shu et al. 2022). Respiration, essential for fish life, can only occur normally in environments with sufficient dissolved oxygen (Lian et al. 2018). However, excessively high levels of dissolved gases can cause fish to suffer from gas bubble disease or even death (Dong et al. 2012; Jiang et al. 2008). In the context of hydropower development, dissolved oxygen levels in reservoirs can become supersaturated due to dam discharge (Wang et al. 2020; Li, Li, et al. 2023; Shen et al. 2015). The average dissolved oxygen levels measured in spring, summer, autumn, and winter were 8.31, 7.72, 8.42, and 9.02, respectively. Combined with the lower water temperatures in autumn and winter, which relatively increase saturation, it is likely that the water body's dissolved oxygen was in a state of supersaturation, leading to a decrease in fish abundance and biomass.

Electrical conductivity showed a high correlation with fish composition but a lower correlation with fish diversity. A previous study indicates that different species and functional groups respond to water conductivity with varying thresholds, both positive and negative (Wu et al. 2019). The composition of species and functional groups is determined by these responses, and changes in conductivity lead to independent changes within these groups, resulting in community alterations.

The FEve index showed a greater correlation with environmental factors, suggesting that environmental factors impose significant constraints on certain fish species or functional groups within the community, warranting further data analysis.

Studies demonstrate that various environmental factors differently influence fish community structure (Littlefair et al. 2021; Mächler et al. 2021). A study used eDNA metabarcoding technology to survey fish diversity in the Yangtze River Estuary and its adjacent waters. CCA analysis results indicated that water temperature, salinity, and dissolved oxygen are the primary environmental factors influencing seasonal variations in fish composition (Zhang, Yoshizawa, et al. 2019). Another study suggests that environmental factors such as water temperature, pH levels, and UV‐B radiation can influence the shedding and degradation rates of eDNA (Strickler et al. 2015). The influence of environmental factors on the actual distribution of fish and eDNA molecules is complex. Therefore, understanding how different environmental factors affect the hydrological characteristics of basins and the rates of eDNA shedding and degradation will help to reveal fish spatial and temporal distribution patterns more comprehensively and accurately. This study discussed the role of six environmental factors, with future research needed to further explore the impact of additional environmental factors on the spatial and temporal distribution of fish in the upper Yangtze River.

## 5 Conclusion

This study conducted a continuous four‐season fish survey in the upper reaches of the Yangtze River using eDNA metabarcoding technology. It provided a multidimensional overview of fish community structure by analyzing species and functional group composition, alpha and beta diversity, functional diversity, taxonomic diversity, and phylogenetic diversity. Additionally, the study explored the impact of environmental factors on the structure of fish communities. The results demonstrate that examining fish communities from the perspective of functional groups facilitates a more straightforward understanding and display of the relationships among fish communities within ecosystems. Multidimensional diversity indices describe the structure of fish communities from various perspectives, some of which are relatively independent while others are interconnected, assisting in the more effective evaluation of biological integrity. These findings contribute important baseline information for understanding fish biodiversity in the upper Yangtze River and provide a scientific basis for the formulation and implementation of conservation policies. Furthermore, it offers practical experience for the further utilization of data from eDNA metabarcoding technology in monitoring fish resources.

## Author Contributions

**Yixiao Li:** data curation (equal), methodology (equal), writing – original draft (equal). **Lei Zhou:** investigation (equal), methodology (equal). **Renhui Luo:** data curation (equal), investigation (equal), software (equal). **Zhiling Dong:** formal analysis (equal), investigation (equal). **Hongsen Lv:** resources (equal), software (equal), writing – review and editing (equal). **Jingning Ling:** validation (equal), writing – review and editing (equal). **Weizhi Yao:** supervision (equal). **Wenping He:** conceptualization (lead), supervision (lead), validation (lead).

## Conflicts of Interest

The authors declare no conflicts of interest.

## Supporting information


**Data S1:** ece372215‐sup‐0001‐DataS1.docx.

## Data Availability

The data that support the findings of this study are available in [Dryad] at [DOI: 10.5061/dryad.rn8pk0pnw], reference number [DOI: 10.5061/dryad]. These data were derived from the following resources available in the public domain: [10.5061/dryad.rn8pk0pnw].
