# Supplementary material for: Seasonal Differences in Fish Community Structure in the Upper Yangtze River Based on eDNA Metabarcoding: A Multi‐Dimensional Analysis
Source: Ecol Evol. 2025 Oct 14;15(10):e72215. doi: 10.1002/ece3.72215 (PMC12521629; doi:10.1002/ece3.72215)
Supplement: Supplementary file 1 — Data S1: ece372215‐sup‐0001‐DataS1.docx. [file ECE3-15-e72215-s001.docx]

**Supplementary Table**

Table S1 Description of functional traits of fish.

| Functional traits | Type of variable | Character description or range of values |
| --- | --- | --- |
| Egg type | Categorical | Sticky Eggs, Drifting Eggs, Demersal Eggs，Floating Eggs, Other Egg Types |
| Migration type | Categorical | Migratory, Sedentary |
| Diet | Categorical | Omnivory, Carnivore, Herbivore, Filter-Feeding |
| Vertical distribution | Categorical | Demersal, Mesopelagic, Pelagic |
| Flow velocity preference | Categorical | Quiet Slow Flow, Rapid Flow, Euroky |
| Body shape | Categorical | Fusiform, Compressed, Depressed, Cylindrical, Qviform, Other Body Shapes |
| Mouth position | Categorical | Inferior, Subinferior, Terminal, Subsuperior, Superior |
| Minimum age of sexual maturity（♂） | Continuous | 0. 17~5 |
| Minimum age of sexual maturity（♀） | Continuous | 0. 17~7 |

Table S2 List of fishes in the upper reaches of the Yangtze River based on eDNA metabarcoding technology.

| Species | Spring | Summer | Autumn | Winter |
| --- | --- | --- | --- | --- |
| I. Acipenseriformes |  |  |  |  |
| i. Acipenseridae |  |  |  |  |
| 1. *Acipenser* *dabryanus*●★ | + | + | + | + |
| II. Cypriniformes |  |  |  |  |
| ii. Balitoridae |  |  |  |  |
| 2. *Jinshaia* *abbreviata*● | + |  |  | + |
| 3. *Sinogastromyzon* *sichangensis* | + |  | + | + |
| 4. *Sinogastromyzon* *szechuanensis*● | + | + |  | + |
| iii. Botiidae |  |  |  |  |
| 5. *Leptobotia* *elongata*●★ | + | + | + | + |
| 6. *Leptobotia* *microphthalma*● | + | + | + | + |
| 7. *Leptobotia* *pellegrini* | + | + |  |  |
| 8. *Leptobotia* *rubrilabris*●★ | + | + |  |  |
| 9. *Leptobotia* *taeniops* | + |  | + |  |
| 10. *Parabotia* *fasciatus* | + | + | + | + |
| 11. *Sinibotia* *reevesae*● | + | + |  |  |
| 12. *Sinibotia* *superciliaris* | + | + | + | + |
| iv. Catostomidae |  |  |  |  |
| 13. *Myxocyprinus* *asiaticus*★ |  |  |  | + |
| v. Cobitidae |  |  |  |  |
| 14. *Cobitis* *sinensis* | + | + |  |  |
| 15. *Misgurnus* *anguillicaudatus* | + | + |  | + |
| 16. *Misgurnus* *mizolepis* |  |  | + |  |
| 17. *Paracobitis* *potanini*● | + | + |  |  |
| 18. *Paracobitis* *variegata* | + | + |  |  |
| 19. *Paramisgurnus* *dabryanus* | + | + | + | + |
| 20. *Triplophysa* *anterodorsalis* | + | + | + | + |
| 21. *Triplophysa* *bleekeri* |  | + |  |  |
| 22. *Triplophysa* *rosa* |  |  | + |  |
| 23. *Triplophysa* *xichangensis* |  |  | + |  |
| vi. Cyprinidae |  |  |  |  |
| 24. *Abbottina* *rivularis* | + | + |  | + |
| 25. *Acanthorhodeus* *chankaensis* | + | + | + | + |
| 26. *Acheilognathus* *barbatulus* | + | + | + |  |
| 27. *Acheilognathus* *macropterus* | + |  | + | + |
| 28. *Acheilognathus* *omeiensis*● |  | + |  | + |
| 29. *Acrossocheilus* *monticola*● | + | + | + | + |
| 30. *Acrossocheilus* *yunnanensis* | + | + |  | + |
| 31. *Ancherythroculter* *wangi*● | + | + | + | + |
| 32. *Belligobio* *nummifer* | + | + | + |  |
| 33. *Carassius* *auratus* | + | + | + | + |
| 34. *Cirrhinus* *cirrhosus*◆ | + | + | + | + |
| 35. *Coreius* *guichenoti*●★ | + |  |  |  |
| 36. *Coreius* *heterodon* | + | + |  | + |
| 37. *Ctenopharyngodon* *idella* | + | + | + | + |
| 38. *Culter* *alburnus* | + | + | + | + |
| 39. *Culter* *dabryi* | + | + | + | + |
| 40. *Culter* *oxycephaloides* |  | + |  | + |
| 41. *Chanodichthys* *erythropterus* | + | + | + | + |
| 42. *Cyprinus* *carpio* | + | + | + | + |
| 43. *Danio* *rerio* | + | + | + | + |
| 44. *Distoechodon* *tumirostris* | + |  | + | + |
| 45. *Elopichthys* *bambusa* | + | + | + | + |
| 46. *Gobiobotia* *filifer* | + | + |  | + |
| 47. *Gobiocypris* *rarus*★ |  | + | + |  |
| 48. *Hemibarbus* *labeo* | + | + | + |  |
| 49. *Hemibarbus* *maculatus* | + | + | + | + |
| 50. *Hemiculter* *bleekeri* | + | + | + | + |
| 51. *Hemiculter* *leucisculus* | + | + | + | + |
| 52. *Hemiculter* *tchangi*● | + | + | + | + |
| 53. *Hemiculterella* *sauvagei*● | + | + | + | + |
| 54. *Hypophthalmichthys* *molitrix* | + | + | + | + |
| 55. *Hypophthalmichthys* *nobilis* | + | + | + | + |
| 56. *Megalobrama* *amblycephala*◆ | + | + | + | + |
| 57. *Megalobrama* *pellegrini*● |  | + |  | + |
| 58. *Microphysogobio* *kiatingensis* | + |  | + | + |
| 59. *Mylopharyngodon* *piceus* | + | + | + | + |
| 60. *Ochetobius* *elongatus* | + | + | + | + |
| 61. *Onychostoma* *simus* | + | + | + | + |
| 62. *Opsariichthys* *bidens* | + | + | + | + |
| 63. *Parabramis* *pekinensis* | + | + | + | + |
| 64. *Platysmacheilus* *nudiventris*● | + | + |  |  |
| 65. *Procypris* *rabaudi*●★ | + | + | + | + |
| 66. *Pseudobrama* *simoni* | + | + | + | + |
| 67. *Pseudohemiculter* *dispar* | + | + | + | + |
| 68. *Pseudolaubuca* *engraulis* | + | + | + |  |
| 69. *Pseudolaubuca* *sinensis* | + | + | + | + |
| 70. *Pseudorasbora* *parva*◆ | + | + | + | + |
| 71. *Rhinogobio* *cylindricus*● | + | + | + | + |
| 72. *Rhinogobio* *typus* | + | + | + | + |
| 73. *Rhinogobio* *ventralis*★ | + |  | + |  |
| 74. *Rhodeus* *ocellatus* | + | + | + |  |
| 75. *Rhodeus* *sinensis* | + | + | + | + |
| 76. *Sarcocheilichthys* *davidi*★ | + | + |  |  |
| 77. *Sarcocheilichthys* *sinensis* | + | + |  | + |
| 78. *Saurogobio* *dabryi* | + | + | + | + |
| 79. *Saurogobio* *dumerili* |  | + | + |  |
| 80. *Schizothorax* *davidi* | + | + | + | + |
| 81. *Sinibrama* *taeniatus*● | + |  |  |  |
| 82. *Spinibarbus* *sinensis* | + | + | + | + |
| 83. *Squalidus* *argentatus* | + | + | + | + |
| 84. *Squaliobarbus* *curriculus* | + | + | + | + |
| 85. *Tinca* *tinca*◆ | + | + | + |  |
| 86. *Folifer* *brevifilis* | + | + | + | + |
| 87. *Toxabramis* *swinhonis* | + | + |  | + |
| 88. *Xenocypris* *davidi* | + | + | + | + |
| 89. *Xenophysogobio* *boulengeri*● | + | + |  |  |
| 90. *Zacco* *platypus* | + | + | + | + |
| III. Cyprinodontiformes |  |  |  |  |
| vii. Adrianichthyidae |  |  |  |  |
| 91. *Oryzias* *sinensis* | + | + | + | + |
| viii. Poeciliidae |  |  |  |  |
| 92. *Gambusia* *affinis*◆ | + | + | + | + |
| IV. Gobiiformes |  |  |  |  |
| ix. Gobiidae |  |  |  |  |
| 93. *Rhinogobius* *cliffordpopei* | + | + | + | + |
| 94. *Rhinogobius* *giurinus* | + | + | + | + |
| V. Perciformes |  |  |  |  |
| x. Centrarchidae |  |  |  |  |
| 95. *Micropterus* *dolomieu* | + | + | + | + |
| 96. *Micropterus* *salmoides*◆ | + | + | + | + |
| xi. Channidae |  |  |  |  |
| 97. *Channa* *argus* | + | + | + | + |
| xii. Cichlidae |  |  |  |  |
| 98. *Oreochromis* spp. ◆ | + | + | + | + |
| xiii. Eleotridae |  |  |  |  |
| 99. *Hypseleotris* *swinhonis* | + | + |  | + |
| xiv. Sinipercidae |  |  |  |  |
| 100. *Siniperca* *chuatsi* | + | + | + | + |
| 101. *Siniperca* *roulei* | + | + | + |  |
| 102. *Siniperca* *scherzeri* | + | + | + | + |
| VI. Salmoniformes |  |  |  |  |
| xv. Salangidae |  |  |  |  |
| 103. *Hemisalanx* *brachyrostralis*◆ | + | + | + | + |
| 104. *Neosalanx* *taihuensis*◆ | + | + | + | + |
| 105. *Protosalanx* *hyalocranius*◆ | + | + | + | + |
| VII. Siluriformes |  |  |  |  |
| xvi. Amblycipitidae |  |  |  |  |
| 106. *Liobagrus* *kingi*★ |  | + |  | + |
| 107. *Liobagrus* *marginatoides*● | + | + | + |  |
| 108. *Liobagrus* *marginatus* | + | + | + | + |
| xvii. Bagridae |  |  |  |  |
| 109. *Hemibagrus* *macropterus* | + | + | + | + |
| 110. *Pelteobagrus* *fulvidraco* | + | + | + | + |
| 111. *Pelteobagrus* *nitidus* | + | + | + | + |
| 112. *Pseudobagrus* *brevicaudatus* | + | + |  |  |
| 113. *Pseudobagrus* *crassilabris* | + | + |  | + |
| 114. *Pseudobagrus* *medianalis* | + | + | + |  |
| 115. *Pseudobagrus* *pratti* | + | + | + | + |
| xviii. Ictaluridae |  |  |  |  |
| 116. *Ictalurus* *punctatus*◆ | + | + | + | + |
| xix. Siluridae |  |  |  |  |
| 117. *Silurus* *asotus* | + | + | + | + |
| 118. *Silurus* *meridionalis* | + | + | + | + |
| xx. Sisoridae |  |  |  |  |
| 119. *Glyptothorax* *fokiensis* | + | + |  |  |
| 120. *Glyptothorax* *sinensis* | + | + |  |  |

Note: "I~VII" stands for orders, "i~xx" for families, "1~120" for species; "★" stands for endangered and rare species, "●" stands for endemic fish in the upper reaches of the Yangtze River, "◆" stands for alien species, "+" stands for the presence of such fish.

Table S3 Fish species included in each functional group

| Functional Group | Species |
| --- | --- |
| FG1 | *Megalobrama pellegrini* |
|  | *Megalobrama amblycephala* |
|  | *Micropterus salmoides* |
|  | *Tinca tinca* |
|  | *Gobiocypris rarus* |
|  | *Hypseleotris swinhonis* |
|  | *Oryzias sinensis* |
|  | *Pseudorasbora parva* |
|  | *Gambusia affinis* |
|  | *Belligobio nummifer* |
|  | *Misgurnus mizolepis* |
|  | *Procypris rabaudi* |
|  | *lctalurus punctatus* |
| FG2 | *Culter alburnus* |
|  | *Cultrichthys erythropterus* |
|  | *Culter oxycephaloides* |
|  | *Culter dabryi* |
|  | *Opsariichthys bidens* |
|  | *Ancherythroculter wangi* |
| FG3 | *Pseudolaubuca sinensis* |
|  | *Hemiculter tchangi* |
|  | *Hemiculter leucisculus* |
|  | *Pseudohemiculter dispar* |
|  | *Hemiculterella sauvagei* |
|  | *Zacco platypus* |
|  | *Toxabramis swinhonis* |
|  | *Danio rerio* |
|  | *Cyprinus carpio* |
|  | *Carassius auratus* |
|  | *Oreochromis* spp. |
|  | *Sarcocheilichthys davidi* |
|  | *Sarcocheilichthys sinensis* |
| FG4 | *Protosalanx hyalocranius* |
|  | *Neosalanx taihuensis* |
|  | *Hemisalanx brachyrostralis* |
|  | *Channa argus* |
|  | *Gobiobotia filifer* |
|  | *Siniperca scherzeri* |
|  | *Siniperca chuatsi* |
|  | *Siniperca roulei* |
| FG5 | *Acheilognathus omeiensis* |
|  | *Acheilognathus macropterus* |
|  | *Acheilognathus barbatulus* |
|  | *Acanthorhodeus chankaensis* |
|  | *Rhodeus sinensis* |
|  | *Rhodeus ocellatus* |
| FG6 | *Rhinogobius giurinus* |
|  | *Rhinogobius cliffordpopei* |
|  | *Micropterus dolomieu* |
|  | *Paracobitis variegata* |
|  | *Paracobitis potanini* |
|  | *Abbottina rivularis* |
|  | *Saurogobio dumerili* |
|  | *Misgurnus anguillicaudatus* |
|  | *Saurogobio dabryi* |
|  | *Microphysogobio kiatingensis* |
|  | *Triplophysa bleekeri* |
|  | *Triplophysa anterodorsalis* |
|  | *Triplophysa rosa* |
|  | *Platysmacheilus nudiventris* |
|  | *Triplophysa xichangensis* |
|  | *Cobitis sinensis* |
|  | *Sinogastromyzon szechuanensis* |
|  | *Sinogastromyzon sichangensis* |
| FG7 | *Distoechodon tumirostris* |
|  | *Paramisgurnus dabryanus* |
|  | *Acrossocheilus yunnanensis* |
|  | *Hemibarbus maculatus* |
|  | *Hemibarbus labeo* |
|  | *Pelteobagrus nitidus* |
|  | *Pelteobagrus fulvidraco* |
|  | *Pseudobrama simoni* |
|  | *Cirrhinus cirrhosus* |
|  | *Folifer brevifilis* |
|  | *Acrossocheilus monticola* |
| FG8 | *Pseudobagrus crassilabris* |
|  | *Pseudobagrus brevicaudatus* |
|  | *Pseudobagrus medianalis* |
|  | *Pseudobagrus pratti* |
|  | *Silurus meridionalis* |
|  | *Silurus asotus* |
|  | *Liobagrus marginatoides* |
|  | *Glyptothorax sinensis* |
|  | *Glyptothorax fokiensis* |
|  | *Hemibagrus macropterus* |
|  | *Liobagrus marginatus* |
|  | *Liobagrus kingi* |
| FG9 | *Leptobotia microphthalma* |
|  | *Leptobotia elongata* |
|  | *Leptobotia pellegrini* |
|  | *Leptobotia rubrilabris* |
|  | *Leptobotia taeniops* |
|  | *Jinshaia abbreviata* |
|  | *Rhinogobio typus* |
|  | *Rhinogobio cylindricus* |
|  | *Rhinogobio ventralis* |
|  | *Xenophysogobio boulenger* |
|  | *iSinibotia superciliaris* |
|  | *Sinibotia reevesae* |
|  | *Schizothorax davidi* |
|  | *Myxocyprinus asiaticus* |
|  | *Xenocypris davidi* |
|  | *Parabotia fasciatus* |
|  | *Spinibarbus sinensis* |
|  | *Onychostoma simus* |
|  | *Acipenser dabryanus* |
| FG10 | *Pseudolaubuca engraulis* |
|  | *Hemiculter bleekeri* |
|  | *Parabramis pekinensis* |
|  | *Sinibrama taeniatus* |
|  | *Ochetobius elongatus* |
|  | *Hypophthalmichthys nobilis* |
|  | *Hypophthalmichthys molitrix* |
|  | *Elopichthys bambusa* |
|  | *Coreius heterodon* |
|  | *Coreius guichenoti* |
|  | *Squalidus argentatus* |
|  | *Squaliobarbus curriculus* |
|  | *Mylopharyngodon piceus* |
|  | *Ctenopharyngodon idella* |
